# Preeruptive Intracoronal Dentin Resorption: Radiographic Diagnosis and Clinical Approach

**DOI:** 10.1155/crid/2799431

**Published:** 2025-10-09

**Authors:** Adrian Gomez-Fernandez, Maria Fernanda Alfaro-Solis

**Affiliations:** Postgraduate Program in Paediatric Dentistry, Faculty of Dentistry, University of Costa Rica, Montes de Oca, San Jose, Costa Rica

**Keywords:** case report, CBCT, occult caries, pediatric dentistry, preeruptive intracoronal resorption

## Abstract

**Background:**

Preeruptive intracoronal resorption (PEIR) is a rare, asymptomatic condition typically detected incidentally on radiographic examination. Its etiology remains unclear, and clinical management is often controversial due to the lack of standardized treatment protocols.

**Case:**

A 14-year-old female was referred after routine orthodontic imaging revealed multiple radiolucent lesions in unerupted teeth. Cone beam computed tomography (CBCT) confirmed a hypodense intracoronal lesion in Tooth 37. Initial management included interdisciplinary assessment, pulp vitality testing, and protective restoration with glass ionomer cement following electrosurgical removal of the distal operculum. Despite early intervention, the tooth developed pulp necrosis, requiring extraction. Histopathological analysis of pericoronal tissue revealed loose connective tissue consistent with follicular origin or a benign odontogenic lesion such as a myxoma.

**Conclusion:**

This case underscores the importance of early diagnosis, advanced imaging techniques, and an interdisciplinary approach in managing PEIR. Tailored treatment strategies based on lesion progression are essential, and long-term monitoring is critical to prevent complications. Further studies are needed to define clear clinical guidelines for PEIR.

## 1. Introduction

Preeruptive intracoronal resorption (PEIR) is an uncommon pathological condition that affects the dentin of unerupted teeth. It is characterized by well-defined radiolucent cavities within the crown, typically located at the dentinoenamel junction, and is detected exclusively through radiographic examinations, as it does not present clinical symptoms prior to eruption [1, 2]. Initially referred to as “occult caries,” the term PEIR is now preferred, as it more accurately reflects its noncarious origin [3, 4].

The first documented case of PEIR was reported in 1941 [4]. Since then, several etiological theories have been proposed, including apical inflammation of primary teeth, dentin developmental defects, dental caries, and ectopic eruption [5, 6]. The most widely accepted hypothesis suggests that resorption results from the invasion of resorptive cells into forming dentin, facilitated by disruptions in crown development [2, 5]. Histologically, these lesions contain soft tissue, inflammatory cells, and osteoclasts, supporting the theory of an active resorptive process distinct from conventional caries [3, 7].

The prevalence of PEIR ranges from 0.2% to 27.3%, depending on the population studied and the diagnostic methods used [8, 9]. Molars and premolars are the most frequently affected teeth, with mandibular second molars and maxillary premolars reported most often [9]. Recent studies have also identified mandibular and maxillary first molars as potential sites, with a prevalence as high as 31% in impacted teeth [5, 10]. Although PEIR has been mainly reported in permanent dentition, isolated cases in primary dentition have also been described, potentially underdiagnosed due to the infrequent use of radiographs in unerupted primary teeth [4].

Since PEIR is asymptomatic before eruption, diagnosis relies entirely on systematic radiographic evaluations. Cone beam computed tomography (CBCT) has proven useful in assessing lesion extent and proximity to the pulp chamber, aiding in treatment planning [10, 11]. The lesion may remain stable or progress rapidly, causing significant structural damage, underscoring the importance of early detection [12].

Despite increasing clinical recognition, there is no consensus on the ideal treatment approach. Some authors recommend monitoring small lesions, while others suggest surgical exposure and restoration or extraction in advanced cases [13, 14]. Biocompatible restorative materials such as glass ionomer cement and Biodentine have been proposed for pulp preservation [3]. This article presents a clinical case of PEIR in a pediatric patient, emphasizing the importance of early diagnosis, interdisciplinary management, and the role of advanced imaging tools.

## 2. Case Report

### 2.1. Medical History

A 14-year-old Costa Rican female, single and a high school student, was referred to the postgraduate program in pediatric dentistry for dental evaluation after an orthodontic assessment revealed radiolucent areas in several unerupted teeth. She had no relevant medical or family history and was not taking any medications. At the time of the consultation, she was asymptomatic and wore fixed orthodontic appliances in the upper arch, which were recommended for removal to facilitate a more accurate diagnosis.

### 2.2. Clinical and Radiographic Findings

**Figure 1:**
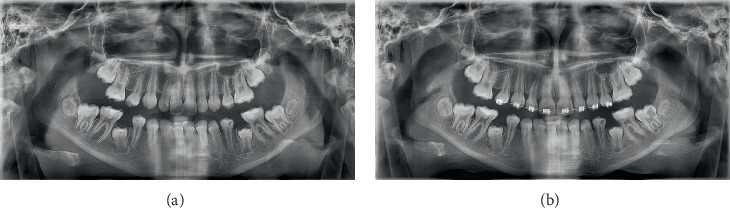
(a) Panoramic radiograph (January 2023): distal radiolucency in Tooth 37 without pulp involvement. (b) Panoramic radiograph (May 2023): rapid lesion progression with potential pulp involvement.

Panoramic radiographs taken in January and May 2023 revealed progressive radiolucency in the mandibular left second molar (Tooth 37) (Figure 1a,b), as well as stable radiolucent areas in Teeth 47, 45, and 34. A periapical radiograph initially raised suspicion of dental caries, although the tooth had not yet erupted.

**Figure 2:**
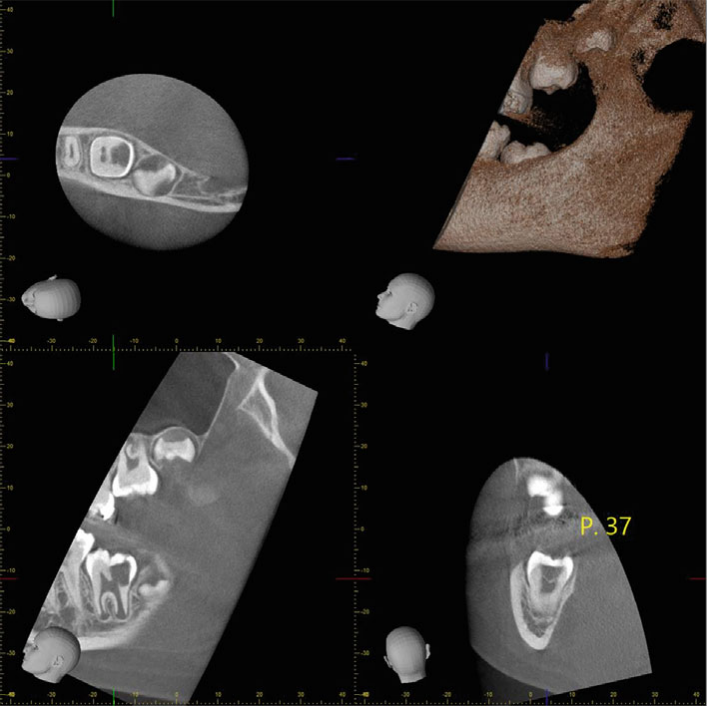
CBCT of Tooth 37: hypodense lesion extending to the pulp chamber, no periapical inflammation.

To confirm the diagnosis and assess the extent of the lesion, a CBCT scan was performed (Figure 2). It showed a hypodense area extending into the pulp chamber, without signs of periapical inflammation. Additionally, a discontinuity in the enamel at the distal fossa was observed.

An interdisciplinary evaluation by the endodontics, orthodontics, and pediatric dentistry departments included pulp vitality testing of Tooth 37, which yielded normal responses at that time. However, the patient reported discomfort in Teeth 26 and 36, prompting occlusal adjustment and the application of fluoride varnish (Clinpro, 3M ESPE). A few days later, she presented with persistent pain in the lower left quadrant.

### 2.3. Treatment

**Figure 3:**
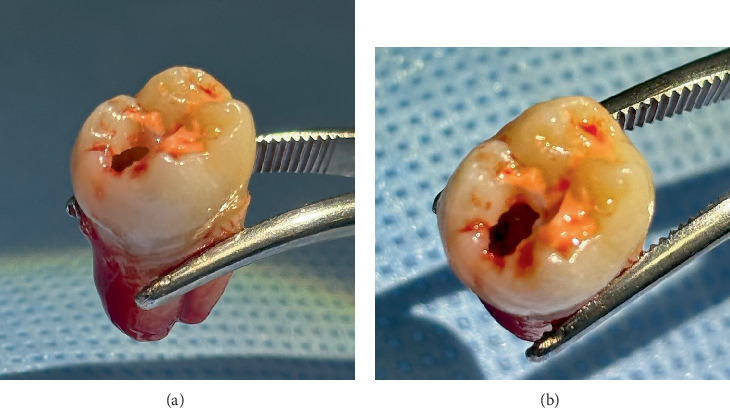
(a) Lingual view of the extracted mandibular left second molar (Tooth 37), showing distal cavitation consistent with intracoronal resorption. (b) Occlusal view of Tooth 37 revealing the enamel discontinuity and exposure of underlying dentin and pulp chamber.

Due to persistent pain, a new endodontic evaluation was conducted in November 2023. Cold testing with EndoIce on Tooth 37 elicited a positive response, indicating potential pulp involvement. A referral to the periodontics department was made for removal of the distal operculum using electrosurgery, which allowed direct visualization of the lesion. A provisional protective restorative material (Fuji TRIAGE, GC Corporation) was then applied. Two days later, during the initiation of endodontic treatment, pulp necrosis was confirmed, and extraction was deemed necessary. The procedure was performed by the oral surgery department. A specimen that included pericoronal soft tissue and, based on clinical inspection, part of the intracoronal lesion contents was collected for histopathological analysis. The extracted mandibular left second molar revealed cavitation consistent with intracoronal resorption (Figure 3). Following extraction, the mandibular left second molar (Tooth 37) was examined macroscopically.

Histopathological analysis revealed loose connective tissue, consistent with either a dental follicle or a benign lesion such as a myxoma.

### 2.4. Follow-Up

**Figure 4:**
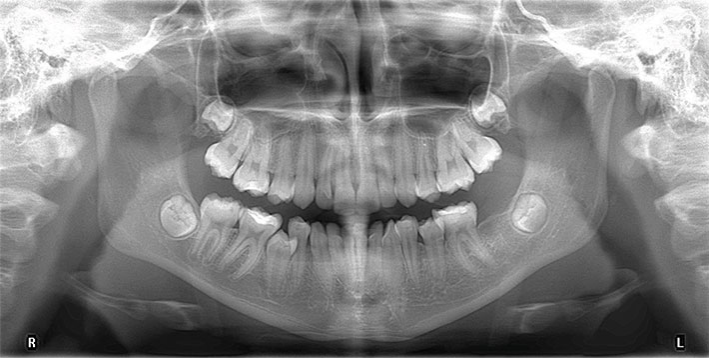
Panoramic radiograph (March 2025).

At the 6-, 10-, and 12-month follow-up appointments, the patient remained asymptomatic. Periapical radiographs showed no new lesions or progression of existing ones. The patient continues to be monitored through interdisciplinary follow-up to assess her long-term clinical progress (Figure 4).

## 3. Discussion

This clinical case highlights the importance of early diagnosis and an interdisciplinary approach in managing PEIR, a rare condition whose timely detection can prevent serious complications. The well-defined radiolucency observed at the dentinoenamel junction was consistent with previously reported cases [9]. CBCT allowed precise assessment of the lesion's extent and its proximity to adjacent structures [10, 11].

Although the reported prevalence of PEIR ranges from 0.2% to 27.3%, its asymptomatic nature and low frequency complicate its identification [6, 12]. Therefore, systematic radiographic assessment of unerupted teeth is strongly recommended [15].

In this case, initial management was conservative, in accordance with current literature recommendations [4, 6]. Interventions such as the removal of the distal operculum and the application of Fuji TRIAGE aimed to preserve the tooth's function. However, the lesion progressed to pulp necrosis, necessitating extraction. This outcome underscores that despite timely intervention, tooth preservation may not always be feasible in advanced cases [9, 10].

The timing of diagnosis plays a pivotal role in determining the most appropriate treatment approach for PEIR. In children between 9 and 11 years of age, extraction combined with strategic orthodontic planning for distal molar substitution may be a favorable option—even when the affected tooth appears salvageable—particularly when long-term prognosis is uncertain. Current clinical guidelines and recent literature emphasize that early detection broadens the therapeutic window, allowing for a more individualized approach ranging from restorative management to extraction with orthodontic space closure, adapted to the patient's stage of dental development and occlusal considerations [16–18].

Histopathological analysis of the lesion in the present case revealed loose connective tissue with mild vascularity and edema, without evidence of inflammation, neoplastic changes, or calcified structures. No odontogenic epithelial rests or enamel organ remnants were identified, but the features were compatible with either a myxoma or dental follicular tissue. Although the clinical impression was that the excisional biopsy included both the pericoronal soft tissue and part of the intracoronal contents, the histopathological report describes the sample as connective tissue compatible with dental follicle or myxoid tissue, without explicitly distinguishing between the pericoronal and intracoronal components. Further studies with targeted sampling could help clarify the histological differences between these regions in PEIR lesions.

Previous studies have reported that these resorptive areas may contain fibrous tissue, dental follicle remnants, myxoid areas, or even granulomatous components [12–14]. Additional recent reports have documented intracoronal cavities consisting of fibrovascular connective tissue with inflammatory infiltrate and odontogenic epithelial remnants, as well as fibrous stroma with occasional inflammatory cells and signs of active resorptive activity, further supporting a cell-mediated resorptive process [19, 20]. However, the exact origin and pathological mechanism of tissue resorption remain poorly understood. Further research into the cellular composition of these lesions may help elucidate their etiology and support more precise clinical management.

Ultimately, this case reinforces the value of CBCT in the accurate diagnosis and treatment planning of PEIR, as well as the necessity of interdisciplinary collaboration. Long-term clinical and radiographic follow-up is essential not only to monitor for recurrence but also to prevent the development of new lesions, ensuring improved outcomes for affected patients.

## 4. Conclusion

This clinical case underscores the importance of systematic radiographic evaluation and the use of advanced diagnostic tools such as CBCT in the detection and management of PEIR. The interdisciplinary approach enabled accurate assessment of the lesion and supported informed clinical decision-making.

While conservative treatment is preferable in early stages, this case demonstrates that extraction may be unavoidable in advanced lesions with pulp involvement to prevent further complications. Moreover, the histopathological analysis reinforces the need for tissue evaluation in complex cases to confirm the diagnosis.

This report contributes to the clinical understanding of PEIR by highlighting the need to establish clearer management protocols and encouraging further research into its etiology, prevalence, and clinical progression. It also emphasizes the importance of long-term follow-up to improve both clinical and functional outcomes in affected patients.

## Data Availability

All data supporting the findings of this study are available within the article.
